# Revisiting Antibiotic Resistance Spreading in Wastewater Treatment Plants – Bacteriophages as a Much Neglected Potential Transmission Vehicle

**DOI:** 10.3389/fmicb.2017.02298

**Published:** 2017-11-21

**Authors:** Rolf Lood, Gizem Ertürk, Bo Mattiasson

**Affiliations:** ^1^Department of Clinical Sciences, Division of Infection Medicine, Lund University, Lund, Sweden; ^2^Department of Biotechnology, Lund University, Lund, Sweden

**Keywords:** bacteriophages, antibiotic resistance, wastewater treatment, antibiotic resistance genes, antimicrobial resistance, WWTP

## Abstract

The spread of antibiotic resistance is currently a major threat to health that humanity is facing today. Novel multidrug and pandrug resistant bacteria are reported on a yearly basis, while the development of novel antibiotics is lacking. Focus to limit the spread of antibiotic resistance by reducing the usage of antibiotics in health care, veterinary applications, and meat production, have been implemented, limiting the exposure of pathogens to antibiotics, thus lowering the selection of resistant strains. Despite these attempts, the global resistance has increased significantly. A recent area of focus has been to limit the spread of resistance through wastewater treatment plants (WWTPs), serving as huge reservoirs of microbes and resistance genes. While being able to quite efficiently reduce the presence of resistant bacteria entering any of the final products of WWTPs (e.g., effluent water and sludge), the presence of resistance genes in other formats (mobile genetic elements, bacteriophages) has mainly been ignored. Recent data stress the importance of transduction in WWTPs as a mediator of resistance spread. Here we examine the current literature in the role of WWTPs as reservoirs and hotspots of antibiotic resistance with a specific focus on bacteriophages as mediators of genetic exchange.

## Introduction

Resistance to antibiotics in clinical bacteria has been documented for several decades (Davies and Davies, 2010). Even for the early antibiotics developed in the 1940s, including penicillin (β-lactam), development and spread of resistance happened almost instantaneously, and the clinical impact was noticeable within a few years (Kong et al., 2010). This rapid development of resistance even to newly developed antibiotics indicates that we are fighting a losing battle. The explosion of multidrug and pandrug resistant strains of a diversity of important bacterial pathogens, including ESBL (extended spectrum beta-lactamase), methicillin/vancomycin resistant *Staphylococcus aureus* (MRSA/VRSA), and pandrug resistant *Acinetobacter baumannii* (Fair and Tor, 2014), seen over the last decade is a further indication that our current attempts to dampen the spread of resistance is not as efficient as needed. While resistance genes for antibiotics exist in small environmental microbial populations even before the clinical usage of those antibiotics, the spread of resistance among human pathogens is not commenced until a selective pressure (e.g., usage of antibiotics) is added – triggering a development and selection for resistant bacteria (Davies and Davies, 2010). Of relevance, not all resistance mechanisms are encoded on mobile genetic elements, but can be chromosomally acquired (e.g., ribosome modifications) or be due to intrinsic antibiotic resistance (Blair et al., 2015). In this review, we will focus on horizontal gene-transfer of antibiotic resistance.

The most commonly described horizontal gene-transfer is conjugation and arguably the most efficient, transferring a plasmid or transposon between bacteria through direct contact. Widely studied in the start of the 1960s researchers for the first time identified the “R-factor” (resistance) and the “F-factor” (sex pili) (Mitsuhashi et al., 1962; Hirota et al., 1966), noticing that bacteria with the R-factor were resistant, and in the presence of the F-factor they could spread the resistance (Hirota et al., 1966). Since then, conjugation has been demonstrated in many environments, and between many different bacteria, stressing its relevance in the spread of antibiotic resistance (Davies, 1994). In Gram-negative bacteria, and also in many Gram-positive bacteria, conjugation is mediated through a type 4 secretion system (T4SS) (Shintani et al., 2015), though other systems, including FtsK homologs have been implied in the conjugation of Gram-positive bacteria (Grohmann et al., 2003). The homology of T4SS, or the relaxase protein, forms a method to divide conjugation plasmids into different Mating Pair Formation (MPF) and mobility (MOB) groups, respectively (Smillie et al., 2010). The extensive worldwide usage of antibiotics continually adds a selective pressure onto these strains to maintain and spread the resistance plasmids within the population.

While several attempts have been made to reduce the use of antibiotics, prescribing less antibiotics for seemingly minor infections, the global discrepancy of antibiotic usage, both for human usage and for meat production, has limited the impact of such approach (Van Boeckel et al., 2014). The negative effects of resistant bacteria are most noticeable within the healthcare system: an organization where high levels of antibiotics are used, certainly acting as a hotspot for spreading of resistance (Bonten et al., 2001). Several studies have reported the presence of MRSA and VRSA in hospitals, causing severe infections in patients hospitalized for other reasons (Loomba et al., 2010). Much focus has therefore been on reducing the spread of antibiotic resistant bacteria within hospitals, and in general to lower the amount of antibiotics used on a society level to thus reduce both abundance and spread of antibiotic resistance (Fair and Tor, 2014). While these restrictions have lowered the spread of antibiotic resistance, it is fair to say that antibiotic resistance is still rapidly spreading, and it remains to find a better solution for the problem. Suggestions to fundamentally change the usage of antibiotics have been raised (Dickey et al., 2017), as has the usage of alternatives to traditional antibiotics, including antimicrobial peptides, probiotics, phage therapy, and phage endolysins (Fischetti, 2011). While many of these alternatives show promise in theoretical and experimental settings, it remains to be investigated how they can be part of a global strategy to reduce antibiotic resistance spreading. Furthermore, such a change in therapy is likely to also suffer from development of resistance, though possibly at a lower rate as has been indicated for a few substances (Gilmer et al., 2017). Thus, a general approach to limit spread of resistance is of need.

Though likely being the most often thought of hotspot for antibiotic resistance spread, hospitals and healthcare systems are not the only spot where high amounts of bacteria, viruses, and resistance genes are concentrated into small compartments. A recently recognized main player in the spread of resistance genes is wastewater treatment plants (WWTPs) with hundreds of original articles and reviews published during the last decade only (Schlüter et al., 2007; Bouki et al., 2013; Gatica and Cytryn, 2013; Rizzo et al., 2013), and with several of these reviews specifically focusing on the impact of bacteriophages in this milieu (Withey et al., 2005; Muniesa et al., 2011, 2013; Balcazar, 2014).

## Wastewater Treatment Plants as Hotspots for Antibiotic Resistance Spreading

The WWTPs serve the purpose of making polluted water suitable for usage again through different mechanisms depending on the quality of the wastewater (e.g., sewage) and the technology employed in the plant. Many WWTPs also take advantage of the high nutritional value of the biomass leading to sludge used both as biofertilizer in agriculture and as biogas formation during anaerobic digestion. Several national and international organizations regulate the quality of the effluent water and byproducts of WWTPs. However, on a global scale, the inclinations and economy to implement these quality controls vary (Jiang et al., 2013).

The WWTPs have recently started to be considered one of the main hotspots for spreading of antibiotic resistance (Rizzo et al., 2013); the main reason for this being the vast amounts of bacteria and other microbes passing through the plants every day. Not only bacteria are passing through the plants, but so are a diversity of resistance genes in various forms (resistant bacteria able of conjugation, free plasmids/DNA, and phage particles) (Parsley et al., 2010a), enabling a high probability of gene transfer within this bacterial community (Balcazar, 2014; Wintersdorff et al., 2016). Furthermore, the prevalence of subclinical levels of antibiotics, heavy metal ions, and other bacteriocidal factors present in low concentrations in wastewater further increases the selection of resistant strains in this environment (Li et al., 2010), adding to the complexity.

The presence of resistant bacteria in WWTPs has been recognized for some time, and means to reduce their quantity in effluent water is a high priority for the plants. Due to the association of many microbes with solid particles in wastewater, filtration efficiently removes a high portion of bacteria in the water, and more recent usage of high-efficiency membranes have decreased that amount even further (Purnell et al., 2015). However, though efficient, membrane filtration in large scale is expensive, and therefore alternative methods are searched for. Addition of bactericidal treatments, including chlorination and high UV-radiation, further decreases the survivability of bacteria when reaching the final purification steps in the plant (Gatica and Cytryn, 2013). With those mechanisms in place, only a fraction of bacteria survives the treatments and is released into the water. It has been demonstrated that after membrane filtering, less than three colony-forming units (cfu)/L water can be detected, which could be lowered to below level of detection in the presence of chlorination (Purnell et al., 2015).

Wastewater treatment plant bacterial communities can be highly diverse, and constitute several hundreds of different species (Withey et al., 2005), many of them in biofilm states. Even though the removal of resistant bacteria from the water fraction is substantial, a higher dose of resistant bacteria can be identified in the digested sludge, reaching levels up to 10^8^ gene copies/g sludge (Calero-Cáceres et al., 2014), raising the question if it is acceptable using this material as biofertilizer on agricultural fields. However, at least for treated wastewater, data are suggesting that the impact on the soil resistome is limited. Several comparisons with irrigated farms using either freshwater or treated wastewater, with the latter containing a significantly higher abundance of resistant bacteria, did not result in any immediate difference in the soil resistome, possibly due to the resistant bacteria struggling to outcompete the indigenous microbiota in the soil (Gatica and Cytryn, 2013). While the direct effects of spreading a smaller fraction of resistant bacteria through WWTPs may seem negligible, the indirect effects, serving as a reservoir for resistance genes, should not be underestimated.

**FIGURE 1:**
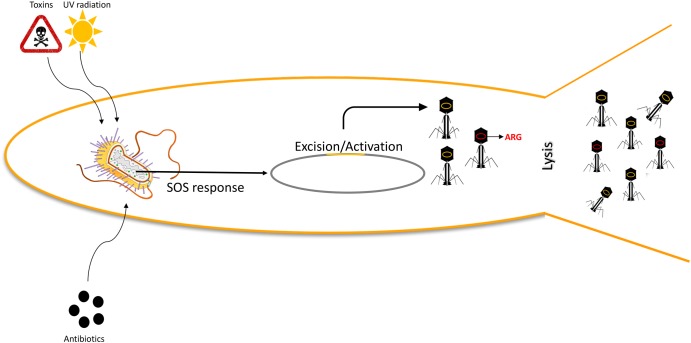
Environmental stress induces transduction events. Environmental factors, including UV light, sub-clinical levels of antibiotics, and other toxic substances will induce SOS responses in bacteria, thus triggering prophage excision (orange) and replication. Phage genetic material will be packaged into the phage particles, as well as random DNA, including Antibiotic Resistance Genes (ARG; red). This increased phage activity will subsequently lead to an increased transduction ratio.

More experimental approaches have been conducted to possibly modulate the WWTP microbiome, rather than specifically address the amounts of bacteria within the plant. Of those approaches, addition of bacteriophages to target specific bacteria (Withey et al., 2005), as well as addition of predatory bacteria targeting Gram-negative bacteria specifically (Feng et al., 2016), has been postulated. While both approaches offer a unique ability to modulate the microbiome, and remove planktonic as well as biofilm bacteria, their impact in spreading of antibiotic resistance has not been studied; neither has the stress they impose upon bacteria. Addition of environmental bacterial stresses, such as those found in WWTPs, is known to trigger transduction (Muniesa et al., 2011; **Figure 1**), why this is of necessity to investigate. Historically, focus has been on removing resistant bacteria, not specifically removing resistance genes, whether existing as free nucleic acids or in phage capsids (Bouki et al., 2013). However, vectors that can deliver resistance are of equal importance, and their fate within WWTPs has just started to unfold (Parsley et al., 2010a).

## The Phageome as a Reservoir of Resistance Genes

Since the independent discovery of bacteriophages in the 1920s by d’Hérelle and Twort, phages have continued to fascinate researchers by their abilities to cause lysis in bacteria and thereby the prospect of using them therapeutically. However, it did not take long before researchers started to realize that phages could not only kill bacteria, but also alter their pathogenicity, lending them virulence while acting as prophages (Canchaya et al., 2004). Furthermore, not only could phages themselves carry virulence genes, but also non-phage DNA could be packaged in phage capsids and injected into other bacteria; a phenomenon known as transduction. The dual function of bacteriophages, being able to both kill bacteria, but possibly also to increase bacterial pathogenicity, has become a dual-edged sword, making it a difficult task to use these potent bacterial killers without the possibility of promoting bacterial genetic exchange.

Bacteriophages have not only been thought of as means to reduce bacterial burden, but have also been used as a tool to estimate the rate of contamination in effluent water from WWTPs since their presence would indicate a lack of removal of pathogenic viruses (e.g., enteroviruses) thus using bacteriophages as a proxy for pathogenic viruses (Mandilara et al., 2006; Yahya et al., 2015). This approach, while being theoretically relevant, has proven to be of limited value, since the removal efficiency of different phages and viruses in WWTPs differs significantly due to the phages’/viruses’ distinct interactions with solids in the wastewater (Purnell et al., 2015). Still, due to the focus on removing mammalian viruses, the methods implemented have been able to also reduce the amounts of viable phages in the effluent water (Haun et al., 2014; Pouillot et al., 2015), while less so in the sludge (Ulbricht et al., 2014). However, the presence of bacteriophages in WWTPs, and the importance of the genetic material they carry in these systems, has not been recognized until recent years (Muniesa et al., 2011). Thus, while the presence of resistance genes in phages has been documented, few articles have focused on the phages themselves as important vectors for resistance. This is of importance, since the resistance genes found in bacteria are reflected in the gene pool found in phages – the phageome – just at a lower ratio (Calero-Cáceres et al., 2014). Thus, while focusing on removal of resistant bacteria in WWTPs, bacteriophages may easily be overlooked, and maintain the resistance genes, being able to spread those genes to new bacteria through transduction.

The viral abundance in WWTPs is extraordinary high, with more than 1000 unique viral genomes identified within the sludge; several of those being bacteriophages (Parsley et al., 2010b). While viruses are too small to be removed by conventional filter systems designed for bacterial removal, their association with bacteria and solids still allows for a high removal grade of viruses through filtration (Purnell et al., 2015). Most wastewater entering the plant contains a bacteriophage load of 10^5-8^ plaque forming units (pfu)/L (Hantula et al., 1991; Carducci and Verani, 2013; Yahya et al., 2015; McMinn et al., 2017). Through conventional treatments, those numbers can be reduced by more than five logarithmic units in the effluent water, while being less affected in the activated sludge (Ulbricht et al., 2014; Purnell et al., 2015). While currently being too costly to implement for treatment of bulk quantities of wastewater (Shahmansouri and Bellona, 2015), application of membrane filtration and reverse osmosis have been proven to significantly increase the reduction in viral particles by several logarithms (Purnell et al., 2015), indicating that future improvement and cost-reduction in this field could prove of importance. However, only specific phages (e.g., coliphages) have been evaluated with these methods, and have been shown to be more sensitive to filtration than the majority of other phages (Jebri et al., 2016). While chlorination is efficient in handling bacterial contamination, bacteriophages are not affected by the chemical and will remain infectious even after long-term treatment (Rizzo et al., 2013). However, other chemical treatments including ozone and Fenton reactions have proven higher efficiency against viral particles. Thus, even though certain of the currently implemented treatments also target viruses, the specific impact on phages is low (Rizzo et al., 2013). The importance of this is further stressed by the high genetic exchange within WWTPs, being facilitated by bacteriophages (Kenzaka et al., 2010; Wintersdorff et al., 2016).

Though being a quite unfavorable mechanism of exchanging DNA, transduction is common in a WWTP setting, and its importance in these systems has recently come in focus. Historically, conjugation has been thought of as the main mechanism of genetic exchange, mainly due to the transfer of a plasmid, thus not necessitating any chromosomal recombination events or replication problem. However, due to the high abundance of microbes, both bacteria and bacteriophages, in an environment with a high concentration of antibiotic resistance genes, transduction has proven important. A common ratio of transduction lies within once every 10^7-9^ bacterial/phage interaction (Muniesa et al., 2013). However, due to the high concentration of microbes in WWTPs, thousands of transductions happen every hour (Rizzo et al., 2013), stressing the necessity of also controlling this path of resistance spreading in WWTPs.

An important aspect of transduction is that even though bacteriophage replication and lysis of bacteria may be limited to certain species, or even down to certain strains, the actual binding to, and injection of genetic material by the phage, displays a much broader spectrum, enabling phages to move genetic material between distinct species (Kenzaka et al., 2010). A common phenotypic development after exposure to high levels of phages is selection of bacteriophage resistant bacteria, mostly due to altered surface structures and thus loss of binding (Labrie et al., 2010). Such phenotypic changes would also influence the rate of transduction negatively, due to limited genetic transfer. However, even though there is a high abundance of phages in wastewater and a replicating phage population, no specific phage resistance has been seen among the bacteria there, likely due to the phage “cocktail” they are exposed to within the WWTPs (Hantula et al., 1991).

**FIGURE 2:**
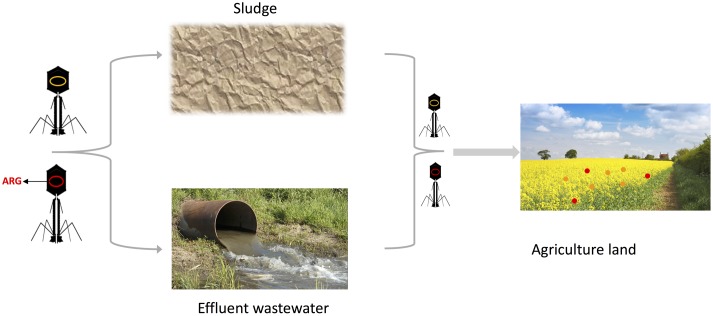
Bacteriophage transmission of resistance genes in wastewater treatment plants (WWTPs). Bacteriophage particles loaded with Antibiotic Resistance Genes (red) will spread through effluent wastewater and sludge. The bacteriophages will encounter the natural soil microbiota and transduce those with their genetic material, rendering a proportion resistant to antibiotics (red circles), while others will not be affected (orange circles). By doing so, the bacteriophages take part in spreading antibiotic resistance, creating a reservoir of genes. Certain figures are derived from the public website www.cronodon.com.

Further adding, and contributing, to the problematic nature of an increased incidence of transduction events in WWTPs is the continuous exposure of environmental stresses for the bacteria. Sub-clinical levels of antibiotics, UV-radiation, toxic substances, and heavy metal ions present in wastewater will lead to an induction of prophages (Motlagh et al., 2015), further increasing the chance of gene transfer. Not only will these toxic substances lead to a general increase in transduced DNA, but specifically in virulent traits, including antibiotic resistance genes. A recent *in vivo* study in mice demonstrated that the exposure of low levels of antibiotics to the animals increased both the actual quantity and the ratio of virulence genes, or adaptation genes – genes allowing for survival in different environments (Modi et al., 2013). Further, selection using one antibiotics (e.g., ampicillin) leads not only to amplification of ampicillin resistance in the phageome but also to other antibiotic resistance traits, functioning as a reservoir of resistance and adaptation genes for the bacteria to take advantage of once the selection is removed. Bacteriophages have been detected carrying resistance genes for among others β-lactams, tetracycline, ampicillin, erythromycin, gentamicin, and quinolones (Balcazar, 2014; Colomer-Lluch et al., 2014a,b). Spreading of these viral particles onto agricultural land, through either wastewater or sludge, will enable sensitive bacteria to take up resistance and virulence genes and mediate these genes to commensal and pathogenic bacteria, thus reducing the effect of the WWTPs in limiting the spread of antibiotic resistance (**Figure 2**).

## Concluding Remarks

While current technologies implemented in WWTPs are efficient in removing most bacterial species and lessen the resistance load among those microbes, it is evident that bacteriophages survive the process; able to infect the microbiota, inject resistance genes, and establish a reservoir of antibiotic resistance genes – a veritable smorgasbord for pathogens and the general microbiota. Membrane bioreactors, ultrafiltration, and the attachment of phages to biosolids within the reactor hold promises to control not only resistant bacteria, but also bacteriophages (Purnell et al., 2015, 2016). Thus, future implementations of the above-mentioned, as well as novel, techniques in WWTPs should not only focus on limiting spread of resistant bacteria, but also limit the survival of bacteriophages and free DNA.

## Author Contributions

RL drafted the initial manuscript. RL, GE, and BM constructively advised on improvements concerning the science and general outline of the review. All authors read and approved the final version of the manuscript.

## Conflict of Interest Statement

The authors declare that the research was conducted in the absence of any commercial or financial relationships that could be construed as a potential conflict of interest.
